# Supplementary material for: Simulating bout-and-pause patterns with reinforcement learning
Source: PLoS One. 2020 Nov 12;15(11):e0242201. doi: 10.1371/journal.pone.0242201 (PMC7660465; doi:10.1371/journal.pone.0242201)
Supplement: Attachment — Submitted filename: Response_to_Reviwers.pdf [file pone.0242201.s001.pdf]

Decision Letter

PONE-D-20-18275

Simulating bout-and-pause patterns with reinforcement learning

PLOS ONE

Dear Dr. Yamada,

Thank you for submitting your manuscript to PLOS ONE. After careful consideration, we feel that it has merit but does not fully meet PLOS ONE's publication criteria as it currently stands. Therefore, we invite you to submit a revised version of the manuscript that addresses the points raised during the review process.

The manuscript should be revised for clarity of the presentation and appropriate references should be discussed and appropriately acknowledged.

Please, provide a statistical description of the three-state Markov model. The transition matrix could be analytically computed in at least some limit cases. That could give the authors some indications regarding the limitations of their model (besides the limitations mentioned in the general discussion of their manuscript).

Reviewers raised concerns about the embedded (hidden) assumptions in the computational model that lead to "emergent" properties. For example, the authors mentioned that "Although our dual model does not explicitly include the bi-exponential model in Eq. (1), IRTs generated by the dual model followed the bi-exponential model." Please, discuss a possibility that the "natural" assumption of a Boltzmann factor in Eq.3 of the model or the logarithmic formula in Eq. 4 does not lead to the "emergent" behavior, such as the bi-exponential model form Eq. 1? How do the authors know that this simple assumption is not the root of the observed bi-exponential and other exciting features?

Pleaser, discuss the potential limitations of the model.

Some important recent papers are missing from discussion and should be included in the revision.

Brackney, R. J., Cheung, T. H. C., & Sanabria, F. (2017). A bout analysis of operant response disruption. Behavioural Processes, 141(Part 1). https://doi.org/10.1016/j.beproc.2017.04.008

Brackney, R. J., & Sanabria, F. (2015). The distribution of response bout lengths and its sensitivity to differential reinforcement. Journal of the Experimental Analysis of Behavior, 104(2), 167–185. https://doi.org/10.1002/jeab.168

Chen, X., & Reed, P. (2020). Factors controlling the micro-structure of human free-operant behaviour: Bout-initiation and within-bout responses are effected by different aspects of the schedule. Behavioural Processes, 175(March), 104106. https://doi.org/10.1016/j.beproc.2020.104106

Daniels, C. W., & Sanabria, F. (2017). About bouts: A heterogeneous tandem schedule of reinforcement reveals dissociable components of operant behavior in Fischer rats. Journal of Experimental Psychology: Animal Learning and Cognition, 43(3), 280–294. https://doi.org/10.1037/xan0000144

Jiménez, Á. A., Sanabria, F., & Cabrera, F. (2017). The effect of lever height on the microstructure of operant behavior. Behavioural Processes, 140, 181–189. https://doi.org/10.1016/j.beproc.2017.05.002

Reed, P. (2015). The structure of random ratio responding in humans. Journal of Experimental Psychology: Animal Learning and Cognition, 41(4), 419–431.

Reed, P., Smale, D., Owens, D., & Freegard, G. (2018). Human performance on random interval schedules. Journal of Experimental Psychology: Animal Learning and Cognition, 44(3), 309–321.

Sanabria, F., Daniels, C. W., Gupta, T., & Santos, C. (2019). A computational formulation of the behavior systems account of the temporal organization of motivated behavior. Behavioural Processes, 169, 103952. https://doi.org/10.1016/j.beproc.2019.103952

Brackney et al. (2017), for instance, report on the effect of various disruptors, including extinction, on bout-organized behavior. Although the distribution of bout lengths is not assessed in the proposed model, it may be important to note that research on that front has been conducted (Brackney & Sanabria, 2015; Jiménez et al., 2017). Also, the proposed model is, in some aspects, comparable to the partially hidden Markov model proposed by Sanabria et al. (2019)—the latter is not a learning model, but accounts for stable-state bi-exponential distribution of IRTs without building that distribution in the model itself.

In page 2, it should be pointed out that the bi-exponential distribution of IRTs has been demonstrated in VI schedules, where reinforcement is available probabilistically at a constant rate. Later in the manuscript the authors make reference to the schedules of reinforcement without explaining them. Also, q does not correspond to the length of a bout but to the *mean* length of a bout.

In page 3, the authors generalize the results from pigeons in Smith et al. (2014) to all animals, when rats actually show a very different pattern. The conclusion they reach is reasonable, assuming that rats engage in alternative behaviors during conditioning.

Page 4, line 5: “both of” should be “both”

Figure 1: Please use a larger font size.

Line 129: “knowledge that is observed” is a strange, ambiguous expression.

Line 170: Do you mean “Fechner’s law”, which implies a representation of magnitude (here, number of lever presses) in logarithmic space. Weber’s law does not imply such representation.

Line 242: “We posit both…” should be “We posit that both…”

Equation 9: Its description includes a parameter b that is not included in the equation.

Line 487: “real animals may have fewer parameters” is a strange expression.v

Please submit your revised manuscript by Oct 16 2020 11:59PM. If you will need more time than this to complete your revisions, please reply to this message or contact the journal office at plosone@plos.org. When you're ready to submit your revision, log on to https://www.editorialmanager.com/pone/ and select the 'Submissions Needing Revision' folder to locate your manuscript file.

Please include the following items when submitting your revised manuscript:

- A rebuttal letter that responds to each point raised by the academic editor and reviewer(s). You should upload this letter as a separate file labeled 'Response to Reviewers'.
- A marked-up copy of your manuscript that highlights changes made to the original version. You should upload this as a separate file labeled 'Revised Manuscript with Track Changes'.
- An unmarked version of your revised paper without tracked changes. You should upload this as a separate file labeled 'Manuscript'.

If you would like to make changes to your financial disclosure, please include your updated statement in your cover letter. Guidelines for resubmitting your figure files are available below the reviewer comments at the end of this letter.

If applicable, we recommend that you deposit your laboratory protocols in protocols.io to enhance the reproducibility of your results. Protocols.io assigns your protocol its own identifier (DOI) so that it can be cited independently in the future. For instructions see: http://journals.plos.org/plosone/s/submission-guidelines#loc-laboratory-protocols

We look forward to receiving your revised manuscript.

Kind regards,

Gennady Cymbalyuk, Ph.D.

Academic Editor

PLOS ONE

Journal Requirements:

When submitting your revision, we need you to address these additional requirements.

1. Please ensure that your manuscript meets PLOS ONE's style requirements, including those for file naming. The PLOS ONE style templates can be found at

https://journals.plos.org/plosone/s/file?id=wjVg/PLOSOne_formatting_sample_main_body.pdf and

https://journals.plos.org/plosone/s/file?id=ba62/PLOSOne_formatting_sample_title_authors_affiliations.pdf

2. Thank you for including your competing interests statement; "The authors have no competing interests."

We note that one or more of the authors are employed by a commercial company: LeapMind Inc.

1. Please provide an amended Funding Statement declaring this commercial affiliation, as well as a statement regarding the Role of Funders in your study. If the funding organization did not play a role in the study design, data collection and analysis, decision to publish, or preparation of the manuscript and only provided financial support in the form of authors' salaries and/or research materials, please review your statements relating to the author contributions, and ensure you have specifically and accurately indicated the role(s) that these authors had in your study. You can update author roles in the Author Contributions section of the online submission form.

Please also include the following statement within your amended Funding Statement.

“The funder provided support in the form of salaries for authors [insert relevant initials], but did not have any additional role in the study design, data collection and analysis, decision to publish, or preparation of the manuscript. The specific roles of these authors are articulated in the ‘author contributions’ section.”

If your commercial affiliation did play a role in your study, please state and explain this role within your updated Funding Statement.

2. Please also provide an updated Competing Interests Statement declaring this commercial affiliation along with any other relevant declarations relating to employment, consultancy, patents, products in development, or marketed products, etc.

Within your Competing Interests Statement, please confirm that this commercial affiliation does not alter your adherence to all PLOS ONE policies on sharing data and materials by including the following statement: "This does not alter our adherence to PLOS ONE policies on sharing data and materials.” (as detailed online in our guide for authors http://journals.plos.org/plosone/s/competing-interests) . If this adherence statement is not accurate and there are restrictions on sharing of data and/or materials, please state these. Please note that we cannot proceed with consideration of your article until this information has been declared.

Please include both an updated Funding Statement and Competing Interests Statement in your cover letter. We will change the online submission form on your behalf.

Please know it is PLOS ONE policy for corresponding authors to declare, on behalf of all authors, all potential competing interests for the purposes of transparency. PLOS defines a competing interest as anything that interferes with, or could reasonably be perceived as interfering with, the full and objective presentation, peer review, editorial decision-making, or publication of research or non-research articles submitted to one of the journals. Competing interests can be financial or non-financial, professional, or personal. Competing interests can arise in relationship to an organization or another person. Please follow this link to our website for more details on competing interests:
http://journals.plos.org/plosone/s/competing-interests

3.We note you have included a table to which you do not refer in the text of your manuscript. Please ensure that you refer to Table 5 in your text; if accepted, production will need this reference to link the reader to the Table.

Response to Editor

Editor's Comment 1: Please, provide a statistical description of the three-state Markov model. The transition matrix could be analytically computed in at least some limit cases. That could give the authors some indications regarding the limitations of their model (besides the limitations mentioned in the general discussion of their manuscript).

In the General Discussion section, we have added a paragraph discussing that the statistical description (i.e., the transition probabilities shown in Figure 3) of the three-state Markov model gives an indication on the limitations of our model. The second paragraph from the last of the revised manuscript reads:
"Fourth, we can design models that are not Markov transition models. The bout-and-pause response patterns shown in Fig. 2 can be generated by a Markov transition model whose transition matrix is given a priori without reinforcement learning. We argue that the statistical description of the Markov model (i.e., the transition matrix defined by the transition probabilities shown in Fig. 3) is not the source of the reproducibility of bout-and-pause patterns. There may be other models that are not formulated by Markov transition, such as the model proposed by McDowell [23]. We can introduce the choice and cost mechanisms to such models."

Also, the transition matrix can be computed as in Table R1.

Table R1. The transition matrix of the three-state Markov model (with both the cost and choice mechanisms).

From \ To	Choice	Operant	Others
Choice	0.0	0.11	0.89
Operant	0.29	0.71	0.0
Others	0.36	0.0	0.64

Editor's Comment 2: Reviewers raised concerns about the embedded (hidden) assumptions in the computational model that lead to “emergent” properties. For example, the authors mentioned that “Although our dual model does not explicitly include the bi-exponential model in Eq. (1), IRTs generated by the dual model followed the bi-exponential model.” Please, discuss a possibility that the “natural” assumption of a Boltzmann factor in Eq.3 of the model or the logarithmic formula in Eq. 4 does not lead to the “emergent” behavior, such as the bi-exponential model form Eq. 1? How do the authors know that this simple assumption is not the root of the observed bi-exponential and other exciting features?

Thank you for raising the question that how we know the specific form equation used in our model is not the cause of bout-and-patterns. To answer this question, we conducted a simulation with a modified model, where the Boltzmann factor and the logarithmic formula were replaced as follows.

- Use the matching law $p_i = Q_i / \sum_i Q_i$ instead of the Boltzmann-type softmax function.
- Use square root instead of logarithm.

The result is shown in Fig. R1 below, which looks similar to Fig. 4(a). It implies that specific forms of equations such as the Boltzman factor in Eq. (3) and the logarithm in Eq. (4) are not the cause of bout-and-pause patterns. The second-to-last paragraph of the "Discussion: Simulation 1" section now has a new sentence: "The specific equation forms such as the softmax function Eq. (3) or the logarithm in Eq. (4) can also be replaceable with other forms."

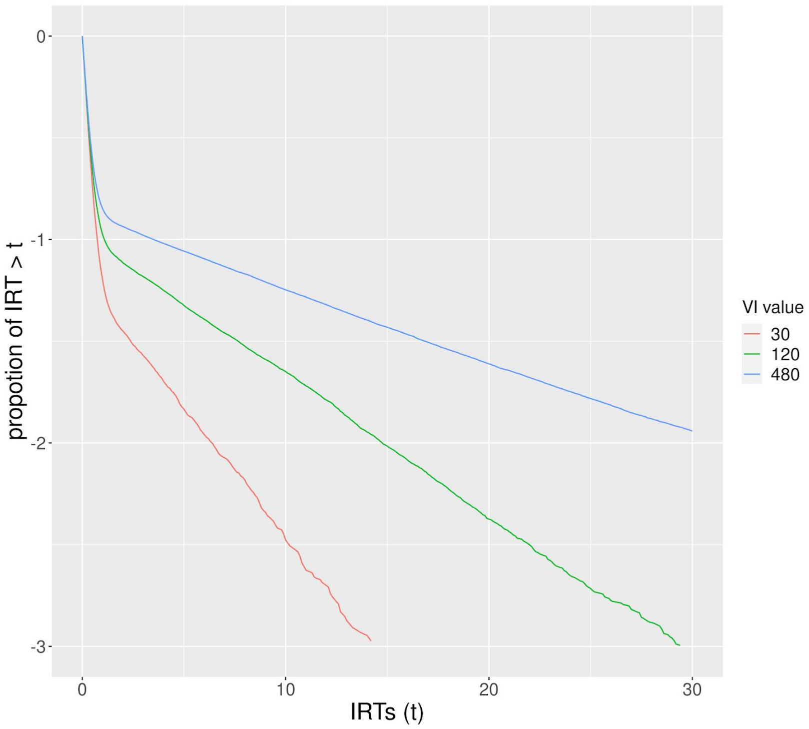

Fig. 1R Log-survivor plots of IRTs generated by the modified model under VI 30 s, VI 120 s, and VI 480 s schedules.

Editor's Comment 3: Pleaser, discuss the potential limitations of the model.

As described in our response to Editor's Comment 1, we have added discussion on the limitation and extendability of the model. The concern raised in Editor's Comment 2 on specific equation forms was found not to be a fundamental limitation of the model since replacing the specific forms did not change the simulation results.

Editor's Comment 4: Some important recent papers are missing from discussion and should be included in the revision.

Thank you for enumerating recent important papers we missed in the previous manuscript. We referred all of them from appropriate locations of the revised manuscript.

Editor's Comment 5: Brackney et al. (2017), for instance, report on the effect of various disruptors, including extinction, on bout-organized behavior. Although the distribution of bout lengths is not assessed in the proposed model, it may be important to note that research on that front has been conducted (Brackney & Sanabria, 2015; Jiménez et al., 2017). Also, the proposed model is, in some aspects, comparable to the partially hidden Markov model proposed by Sanabria et al. (2019)—the latter is not a learning model, but accounts for stable-state bi-exponential distribution of IRTs without building that distribution in the model itself.

Thank you for pointing out the importance of the research front issues. We have added a discussion on this point to General Discussion in our revised manuscript. The second-to-last paragraph of General Discussion of the revised manuscript reads:
"Third, we can assess the plausibility of our model in more detail by conducting simulation under new experimental manipulations including disruptors or analyzing measures that we did not analyze. For example, recent studies showed that the distribution of bout lengths is sensitive to experimental manipulations [13, 33, 34]. Sanabria et al. [35] have proposed a computational formulation of behavior systems [36] and their descriptive model well described bout-and-pause patterns including the distribution of bout lengths."

*Editor's Comment 6: In page 2, it should be pointed out that the bi-exponential distribution of IRTs has been demonstrated in VI schedules, where reinforcement is available probabilistically at a constant rate. Later in the manuscript the authors make reference to the schedules of reinforcement without explaining them. Also, q does not correspond to the length of a bout but to the *mean* length of a bout.*

Thank you for pointing out our insufficiencies on the explanation about VI schedules. In the third paragraph of Introduction, we added a brief description of VI schedules and specified bout-and-pause patterns are observed under this schedule. Also, we have inserted "mean" to the description of q.

Editor's Comment 7: In page 3, the authors generalize the results from pigeons in Smith et al. (2014) to all animals, when rats actually show a very different pattern. The conclusion they reach is reasonable, assuming that rats engage in alternative behaviors during conditioning.

In the sixth paragraph of Introduction, we specified that rats also engage alternative behaviors (i.e. schedule induced behavior, interim behavior, or adjunctive behavior) during conditioning. The added sentence is: "Similar observations have been made for rats, assuming that they engage in alternative behaviors during conditioning [20]."

Editor’s Comment 8:
Page 4, line 5: “both of” should be “both”
Figure 1: Please use a larger font size.
Line 129: “knowledge that is observed” is a strange, ambiguous expression.
Line 170: Do you mean “Fechner’s law”, which implies a representation of magnitude (here, number of lever presses) in logarithmic space. Weber’s law does not imply such representation.
Line 242: “We posit both…” should be “We posit that both…”

Equation 9: Its description includes a parameter b that is not included in the equation.
Line 487: “real animals may have fewer parameters” is a strange expression.

We have fixed all of these points.

Modifications not based on the editor's comments

We noticed that an unnecessary sentence was mistakenly left in the original manuscript at the second paragraph of General Discussion. Thus, we have removed it in the revised manuscript. Removed sentence: "This is an example of the first advantage of reinforcement learning discussed in the next paragraph."

Although Table 5 was not referred to in the previous manuscript, now in the revised manuscript it is referred to in the second-last paragraph of Result and Discussion of Simulation 2.

Response to Reviewers

Reviewer 1

Reviewer 1's Comment 1: I only have two relatively minor concerns regarding the manuscript. First, although the authors provide a useful synthesis of the literature on the microstructure of instrumental behavior, many important recent papers are missing from that synthesis, which makes it appear outdated. Below I have listed several recent papers that the authors omitted, and that I believe would inform the assessment of their model. Because I am co-author in many of these papers, I am disclosing my name in the signature, and my recommendation will not change whether or not the authors choose to include any of them.

Thank you for suggesting recent important articles, which are now referenced from the revised manuscript (also in response to *Editor’s Comments 4*).

Reviewer 1's Comment 2: Brackney et al. (2017), for instance, report on the effect of various disruptors, including extinction, on bout-organized behavior. Although the distribution of bout lengths is not assessed in the proposed model, it may be important to note that research on that front has been conducted (Brackney & Sanabria, 2015; Jiménez et al., 2017). Also, the proposed model is, in some aspects, comparable to the partially hidden Markov model proposed by Sanabria et al. (2019)—the latter is not a learning model, but accounts for stable-state bi-exponential distribution of IRTs without building that distribution in the model itself.

Please see our response to *Editor’s Comment 5*.

Reviewer 1's Comment 3: The second concern is about style—not nearly as important as content, which is excellent in this paper, but it is important nonetheless. In various parts, the manuscript would benefit from economy of expression, precision, clearer

organization of key claims in separate paragraphs, and a more deliberately logical connection between ideas. Below I just point at some salient examples:

*In page 2, it should be pointed out that the bi-exponential distribution of IRTs has been demonstrated in VI schedules, where reinforcement is available probabilistically at a constant rate. Later in the manuscript the authors make reference to the schedules of reinforcement without explaining them. Also, q does not correspond to the length of a bout but to the *mean* length of a bout.*

Please see our response to *Editor's Comment 6*.

Reviewer 1's Comment 4: In page 3, the authors generalize the results from pigeons in Smith et al. (2014) to all animals, when rats actually show a very different pattern. The conclusion they reach is reasonable, assuming that rats engage in alternative behaviors during conditioning.

Please see our response to *Editor's Comment 7*.

Page 4, line 5: "both of" should be "both"

Figure 1: Please use a larger font size.

Line 129: "knowledge that is observed" is a strange, ambiguous expression.

Line 170: I believe the authors mean "Fechner's law", which implies a representation of magnitude (here, number of lever presses) in logarithmic space. Weber's law does not imply such representation.

Line 242: "We posit both…" should be "We posit that both…"

Equation 9: Its description includes a parameter b that is not included in the equation.

Line 487: "real animals may have fewer parameters" is a strange expression.

Please see our response to *Editor's Comment 8.*

Reviewer 2

Reviewer 2's Comment 1: Briefly, the beauty of the model is its parsimony. The authors considered that the bout-and-pause patterns could be captured by a three-state Markov model controlled by two independent mechanisms: (1) the choice between Operant and Others, and (2) the cost in the changeover of behaviors.

At the same time, a three-state Markov is amenable to at least a basic statistical description, and the authors did not attempt that. The transition matrix could be analytically computed in at least some limit cases. That could give the authors some

indications regarding the limitations of their model (besides the limitations mentioned in the general discussion of their manuscript).

Please see our responses to *Editor's Comment 2* and *3*.

Reviewer 2's Comment 2: The second concern I have is about the embedded (hidden) assumptions in the computational model that lead to "emergent" properties. For example, the authors mentioned that "Although our dual model does not explicitly include the bi-exponential model in Eq. (1), IRTs generated by the dual model followed the bi-exponential model." My question is: how do they know that the "natural" assumption of a Boltzmann factor in Eq.3 of the model or the logarithmic formula in Eq. 4 does not lead to the "emergent" behavior, such as the bi-exponential model form Eq. 1? I understand that everybody used Boltzmann's factor in every field of science, but still – how do the authors know that this simple assumption is not the root of the observed bi-exponential and other exciting features?

Please see our response to *Editor's Comment 1*.
